# Pseudo-two-dimensional multiphysics modeling of mass transport and pseudo-enzymatic kinetics in Ti_3_C_2_T_*x*_@Pt MXene-based glucose biosensors

**DOI:** 10.1039/d5ra09807f

**Published:** 2026-02-26

**Authors:** Mohamed Abu Shuheil, Thamer Hani, Roopashree R, Subhashree Ray, Baraa Mohammed Yaseen, Kavitha V, Renu Sharma, Aashna Sinha, Amir Arsalanirad

**Affiliations:** a Faculty of Allied Medical Sciences, Hourani Center for Applied Scientific Research, Al-Ahliyya Amman University Amman Jordan; b Department of Dental Medicine, College of Dental Medicine, AL-Turath University Baghdad Iraq; c Department of Chemistry and Biochemistry, School of Sciences, JAIN (Deemed to be University) Bangalore Karnataka India; d Department of Biochemistry, IMS and SUM Hospital, Siksha ‘O' Anusandhan Bhubaneswar Odisha-751003 India; e Department of Medical Laboratory Technics, College of Health and Medical Technology, Alnoor University Mosul Iraq; f Department of Chemistry, Sathyabama Institute of Science and Technology Chennai Tamil Nadu India; g Department of Chemistry, University Institute of Sciences, Chandigarh University Mohali Punjab India; h School of Applied and Life Sciences, Division of Research and Innovation, Uttaranchal University Dehradun Uttarakhand India; i Young Researchers and Elite Club, Islamic Azad University of Tehran Tehran Iran amirarsalaniradacademic@gmail.com

## Abstract

Accurate glucose sensing in nanozyme-based platforms is fundamentally governed by the coupled interplay between mass transport and surface-confined catalytic reactions, particularly in systems characterized by intrinsic nanoscale heterogeneity. In this work, a pseudo-two-dimensional (pseudo-2D) multiphysics modeling framework is developed to elucidate diffusion-reaction interactions in Ti_3_C_2_T_*x*_@Pt MXene-based glucose biosensors by explicitly resolving two-dimensional diffusion of glucose and hydrogen peroxide in the electrolyte while confining pseudo-enzymatic reactions to laterally heterogeneous Pt catalytic domains described by Michaelis–Menten kinetics. The simulations demonstrate that non-uniform Pt site distributions induce pronounced local substrate depletion, lateral concentration gradients, and an effective thickening of the diffusion layer, resulting in transport bottlenecks that are not captured by conventional one-dimensional models. As a consequence, the pseudo-2D framework predicts a systematic reduction in effective glucose flux, premature saturation of the sensor response, and significant shifts in apparent kinetic parameters, including an increased effective Michaelis constant and a decreased maximum reaction rate, despite identical mean catalyst loading. In addition, the model reveals enhanced accumulation and delayed transport of hydrogen peroxide within the diffusion layer, directly modulating colorimetric signal intensity and response dynamics. Quantitative comparison with experimentally reported UV-vis absorbance spectra and electrochemical response trends shows excellent agreement across physiologically relevant glucose concentrations, confirming the predictive capability of the proposed approach. Overall, these findings highlight the critical role of lateral catalyst dispersion in governing mass transport limitations, apparent kinetics, and sensing performance, and establish the pseudo-2D multiphysics framework as a computationally efficient and physically rigorous tool for the rational design and optimization of heterogeneous MXene nanozyme-based glucose biosensors.

## Abstract 2

Pseudo-2D modeling shows Pt heterogeneity governs mass transport, kinetics, and performance of Ti_3_C_2_T_*x*_@Pt glucose biosensors.

## Graphical abstract

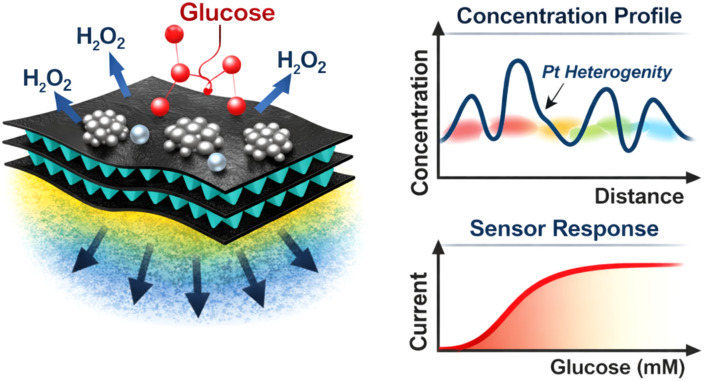

## 1. Introduction

Understanding and optimizing glucose sensing mechanisms remain central to advancing biosensor technologies for clinical diagnostics and health monitoring. Accurate glucose detection is essential in managing metabolic disorders, particularly diabetes mellitus, which continues to present significant global health challenges.^1^ Among the diverse strategies explored, nanozyme-based sensors (artificial enzyme mimetics exhibiting catalytic functionalities) have emerged as robust alternatives to natural enzymes due to their enhanced stability, tunable activity, and cost-effectiveness.^2–4^ Peroxidase-like (POD) nanozymes, especially, catalyze the decomposition of hydrogen peroxide (H_2_O_2_) and generate detectable signals *via* colorimetric, electrochemical, or chemiluminescent pathways.^5,6^ This attribute underlies their broad application in biosensing, including glucose analysis, where glucose oxidase (GO_*x*_) catalyzes glucose oxidation to gluconic acid and H_2_O_2_, which is subsequently processed by the nanozyme.^7^

Two-dimensional (2D) transition metal carbides/nitrides (MXenes) (particularly Ti_3_C_2_T_*x*_) represent a transformative class of materials in nanozyme design and advanced biosensing due to their high conductivity, large surface area, hydrophilic surface terminations, and facile surface functionalization.^8,9^ These features promote efficient electron transport and abundant active sites, which are key for enhancing catalytic efficiency and lowering detection limits.^10,11^ MXene-based composites integrated with noble metal nanoparticles (*e.g.*, Pt, Au) have shown remarkable peroxidase-like activity and improved electrochemical responses for glucose detection, achieving wide linear ranges and low limits of detection (LOD).^12,13^

Despite these material advances, sensor performance is fundamentally governed by mass transport dynamics and heterogeneous catalytic interactions at the nanozyme interface. Traditional modeling techniques often employ one-dimensional (1D) assumptions of homogeneous surfaces and uniform activity,^14^ which inadequately capture the complex interplay between lateral heterogeneity of active sites, diffusion gradients, and reaction kinetics. Recent reports emphasize that non-uniform distributions of catalytic clusters and nanozyme morphology can drastically influence substrate accessibility and effective kinetic parameters, highlighting the limitations of simplified models in predicting sensor behavior.^15,16^

To bridge this gap, multiphysics modeling approaches have been developed to resolve coupled mass transport and surface reaction phenomena in more realistic geometries.^17–19^ For example, mesoscopic studies integrating microkinetic frameworks and transport effects elucidate how catalyst roughness and lateral heterogeneities affect selectivity and reaction pathways in related electrocatalytic systems.^20^ Extending such modeling to nanozyme-based glucose sensing platforms offers critical insight into how diffusion-limited transport and pseudo-enzymatic kinetics collectively govern sensor outputs, such as apparent Michaelis–Menten constants (*K*_m_) and maximum reaction rates.^21,22^

The pseudo-two-dimensional (pseudo-2D) multiphysics model adopted here explicitly accounts for diffusion in both lateral and vertical directions above a heterogeneous Ti_3_C_2_T_*x*_@Pt nanozyme-modified surface.^23^ By confining catalytic reactions to the solid–liquid boundary and allowing two-dimensional diffusion in the overlying domain, the model effectively captures surface clustering effects without resorting to computationally expensive full 3D simulations. Such an approach has been shown to reveal significant deviations in mass transport profiles, effective diffusion layer thickness, and aggregated kinetic behavior compared to traditional 1D formulations.^24–26^ These deviations are especially pronounced when lateral gradients, induced by heterogeneous distributions of active Pt sites, give rise to transport-controlled flux limitation and substrate depletion “hot-spots” that reduce effective substrate availability at the nanozyme surface.

Furthermore, integrating realistic H_2_O_2_ transport and consumption dynamics is essential for colorimetric response prediction, as intermediate buildup and delayed outward diffusion contribute to signal modulation.^27–29^ Similarly, electrochemical reactions (involving direct glucose oxidation at the electrode interface) are sensitive to catalytic site density and transport-limited flux, affecting overall current responses.^30–32^ Consequently, pseudo-2D frameworks that account for spatially varying surface reactivity provide a more accurate mechanistic basis for interpreting experimental calibration curves and for guiding optimization strategies based on nanozyme dispersion and morphology.

In this work, a pseudo-two-dimensional (pseudo-2D) multiphysics model is developed to quantitatively investigate the role of lateral surface heterogeneity on mass transport and pseudo-enzymatic kinetics in a Ti_3_C_2_T_*x*_@Pt nanozyme-based glucose sensing platform. The model, implemented in COMSOL Multiphysics, explicitly couples two-dimensional diffusion of glucose and hydrogen peroxide in the electrolyte with surface-confined Michaelis–Menten reaction kinetics at spatially heterogeneous catalytic domains. Key parameters, including Pt cluster size, inter-cluster spacing, surface coverage, diffusion layer thickness, apparent (*K*_m_), and effective reaction rates, are systematically evaluated. By comparing pseudo-2D predictions with conventional 1D formulations and experimental calibration data, this study aims to elucidate how nanoscale catalyst distribution governs effective kinetic parameters and sensor sensitivity, thereby providing a predictive framework for the rational design and optimization of high-performance nanozyme-based glucose biosensors.

## 2. Methodology

The development of nanozyme-based biosensors for glucose detection relies on the intricate interplay between mass transport phenomena and surface-catalyzed reactions. In this study, a pseudo-two-dimensional (pseudo-2D) multiphysics model is formulated to describe the mass transport and pseudo-enzymatic kinetics of glucose and hydrogen peroxide (H_2_O_2_) in the vicinity of Ti_3_C_2_T_*x*_@Pt MXene nanozymes, as employed in the dual-mode colorimetric and electrochemical glucose detection platform. This modeling approach captures the essential features of heterogeneous surface catalysis while maintaining computational efficiency, making it suitable for elucidating the influence of Pt nanoparticle distribution on sensor performance.

### 2.1. Pseudo-2D geometry

The computational domain is defined as a rectangular two-dimensional region that represents the diffusion layer adjacent to the Ti_3_C_2_T_*x*_@Pt-modified electrode surface. The horizontal dimension (*x*-direction) spans a length *L*, which corresponds to a representative segment of the electrode surface, incorporating variations in the local density and distribution of Pt nanoparticles supported on the Ti_3_C_2_T_*x*_ MXene sheets. The vertical dimension (*y*-direction) extends from *y* = 0 (the active surface) to *y* = *H* (the bulk solution boundary), where *H* denotes the thickness of the diffusion layer.

The lower boundary at *y* = 0 represents the Ti_3_C_2_T_*x*_@Pt nanocomposite surface, where pseudo-enzymatic reactions occur exclusively. The upper boundary at *y* = *H* interfaces with the well-stirred bulk solution, maintaining constant analyte concentrations. Lateral boundaries at *x* = 0 and *x* = *L* are treated as symmetry or no-flux conditions to simulate periodic repetition of the surface features. This pseudo-2D formulation approximates the three-dimensional complexity of the nanocomposite surface by confining reactions to the *y* = 0 boundary while allowing two-dimensional diffusion in the overlying solution layer. The geometry strikes a balance between capturing lateral heterogeneities in catalytic activity and avoiding the prohibitive computational cost of full three-dimensional simulations.

### 2.2. Physics interfaces

The model integrates two primary physics modules: (i) Transport of diluted species in the bulk domain (2D) and (ii) surface reactions at the active boundary. Convection is neglected, as the system operates under quiescent conditions typical of colorimetric assays and chronoamperometric electrochemical measurements, rendering diffusion the dominant transport mechanism. Electrostatic effects and migration are omitted, given the neutral or buffered electrolyte environments used in the experimental protocols.

The species tracked include glucose (Glu) and hydrogen peroxide (H_2_O_2_), the latter being both a product of enzymatic glucose oxidation and a substrate for the peroxidase-like activity of the Ti_3_C_2_T_*x*_@Pt nanozyme. For colorimetric simulations, the oxidation of 3,3′,5,5′-tetramethylbenzidine (TMB) is implicitly incorporated through the H_2_O_2_ consumption rate, whereas electrochemical simulations focus on direct electrooxidation of glucose at the modified electrode.

### 2.3. Governing equations

The pseudo-two-dimensional multiphysics model describes the diffusion-dominated transport of glucose (Glu) and hydrogen peroxide (H_2_O_2_) in the solution domain overlying the heterogeneous Ti_3_C_2_T_*x*_@Pt surface, coupled with surface-confined pseudo-enzymatic reactions. Mass transport for each species *i* (*i* = Glu, H_2_O_2_) is governed by Fick's second law in the two-dimensional domain 33:1
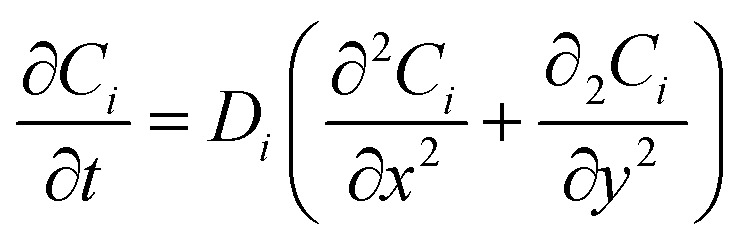
where *C*_*i*_(*x*, *y*, *t*) denotes the local concentration, *t* is time, and *D*_*i*_ is the diffusion coefficient (*D*_Glu_ = 6.7 × 10^−10^ m^2^ s^−1^; *D*_H_2_O_2__ = 1.5 × 10^−9^ m^2^ s^−1^). This equation applies throughout the rectangular domain (0 ≤ *x* ≤ *L*, 0 ≤ *y* ≤ *H*), assuming dilute-solution conditions and negligible convective or migrative contributions, consistent with quiescent experimental setups in both colorimetric and chronoamperometric protocols.^34^

The pseudo-2D approximation is invoked by the scale separation inherent to the system: lateral gradients are significantly weaker than vertical ones in the bulk of the diffusion layer, *i.e.*, |∂*C*_*i*_/∂*x*| ≪ |∂*C*_*i*_/∂*y*|, except in close proximity to the heterogeneous boundary where localized Pt nanoparticle clusters induce modest *x*-directional variations. This enables retention of two-dimensional diffusion while confining catalytic reactions exclusively to the *y* = 0 boundary, avoiding the computational demands of fully three-dimensional heterogeneous modeling.

At the reactive surface (*y* = 0), diffusive flux from the solution phase is balanced by the heterogeneous reaction rate:2
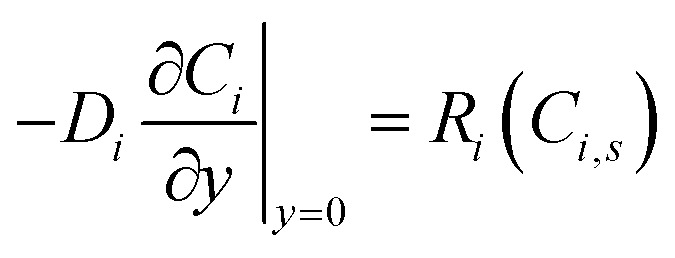
where *C*_*i*_, *s*(*x*, *t*) = *C*_*i*_(*x*, 0, *t*) is the surface concentration, and *R*_*i*_ represents the net consumption (for substrates) or production rate. The peroxidase-like activity of Ti_3_C_2_T_*x*_@Pt, primarily attributed to Pt sites, is described by Michaelis–Menten kinetics for H_2_O_2_ consumption in the colorimetric mode:^35^3
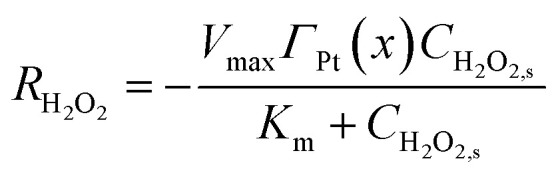
With *V*_max_ the maximum turnover frequency per active site, *K*_m_ the intrinsic Michaelis constant (∼2.5 mM, derived from homogeneous Pt benchmarks adjusted for nanozyme behavior), and *Γ*_Pt(*x*)_ the local surface site density, parameterized sinusoidally (50% amplitude variation around the mean) to emulate clustering observed in TEM/EDS mappings.^34^ Upstream H_2_O_2_ production from glucose oxidase (GOD)-catalyzed glucose oxidation is incorporated as a source term:

4 *R*_H_2_O_2__ = *k*_GOD_*C*_Glu,s_

where *k*_GOD_ is an effective first-order rate constant calibrated to experimental incubation conditions.

For direct glucose electrooxidation in the electrochemical mode, an analogous heterogeneous Michaelis–Menten form is adopted:^35^5
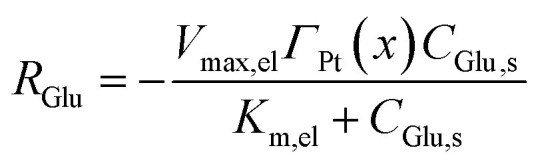
With parameters *V*_max,el_ and *K*_m,el_ tuned to chronoamperometric responses. To extract observable macroscopic quantities comparable to experimental data, spatial averaging along the *x*-direction is performed:6
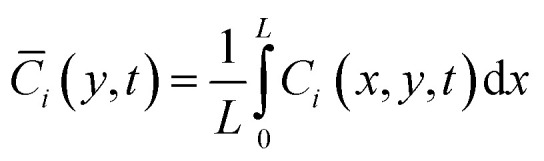


Yielding effective one-dimensional profiles. Similarly, the effective surface concentration *C*^eff^_*i*,s_(*t*) = *C̄*_*i*_(0, *t*) and effective flux 
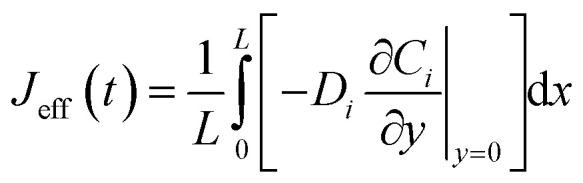
 enable fitting of apparent kinetic parameters (*K*^eff^_m_, *V*^eff^_max_) *via*:^36^7
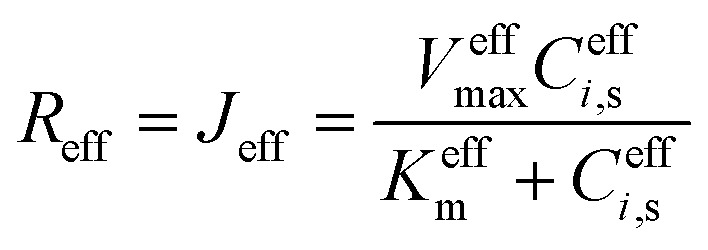


These governing equations, solved *via* finite elements with adaptive meshing near the reactive boundary, provide a mechanistic bridge between nanoscale surface heterogeneity and sensor-level performance metrics, elucidating transport-kinetic interplay without invoking empirical corrections.

For clarity, quantities reported in normalized or relative form (*e.g.*, *V*_max_,eff) are explicitly defined here. Normalized values are obtained by scaling the effective quantity by its corresponding reference value predicted by the uniform 1D model under identical conditions. Specifically, the normalized effective maximum rate is defined as *V*_max_,eff/*V*_max_,1D, where *V*_max_,1D denotes the maximum rate extracted from the conventional one-dimensional uniform-surface model. This normalization is employed to isolate the impact of transport and surface heterogeneity effects from absolute parameter values and to enable direct comparison between modeling frameworks. Using normalized quantities facilitates comparison across models and highlights relative transport-induced penalties, independent of absolute kinetic scaling.

### 2.4. Boundary conditions and initial conditions

At the active surface (*y* = 0), the boundary condition for glucose reflects its consumption in the enzymatic step (colorimetric mode) or electrooxidation (electrochemical mode):8
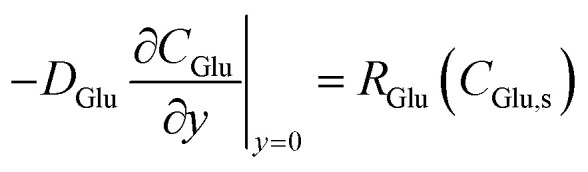


For H_2_O_2_ in the colorimetric pathway, production from glucose oxidase (GOD)-catalyzed oxidation and consumption by the nanozyme are balanced:9
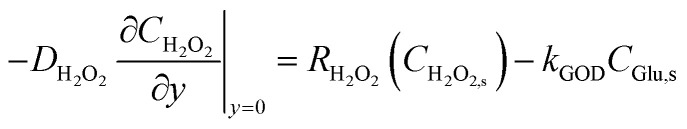
where *k*_GOD_ represents the effective rate of the upstream enzymatic step. At the bulk interface (*y* = *H*), Dirichlet conditions are imposed:

10 *C*_*i*_(*y* = *H*) = *C*_*i*,bulk_

Reflecting the constant reservoir concentrations used in experiments. Lateral boundaries (*x* = 0, *L*) enforce no-flux conditions:11
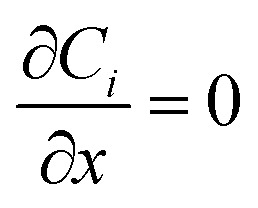


### 2.5. Ensuring conservation and periodicity

Initial conditions assume uniform bulk concentrations throughout the domain at *t* = 0, with the system subsequently evolving toward steady state or transient response depending on the simulated protocol.

The model is implemented in a finite element framework, with mesh refinement near *y* = 0 to resolve steep concentration gradients within the diffusion layer. Steady-state solutions are obtained for concentration-response analyses, while time-dependent simulations capture transient behaviors such as response delays. To facilitate quantitative comparisons, spatially averaged concentrations are computed as:12
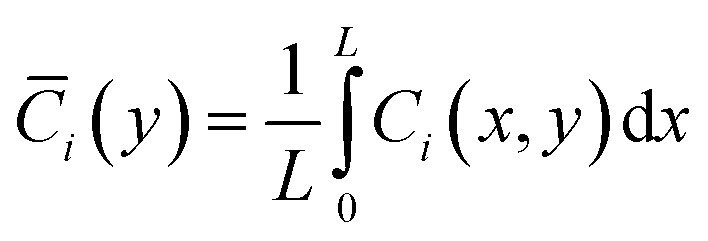


Effective surface concentrations and fluxes are similarly averaged over the *x*-direction:13
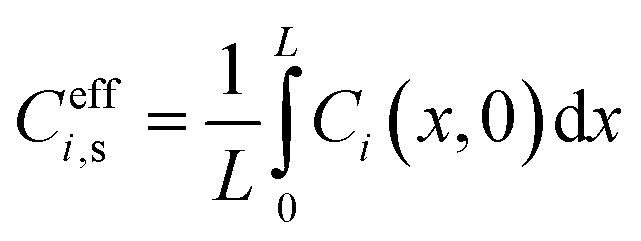
14
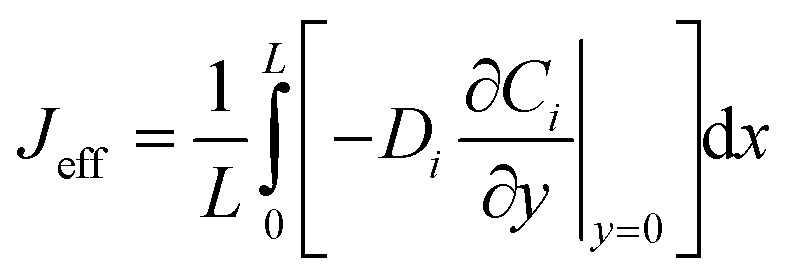


These averaged quantities enable direct extraction of apparent kinetic parameters (*m*^eff^_m_, *V*^eff^_max_) by fitting the effective reaction rate *R*_eff_ = *J*_eff_ to a Michaelis–Menten form.

This methodological framework provides a robust platform for investigating how surface heterogeneity modulates mass transport limitations and apparent catalytic efficiency, offering insights beyond uniform-surface assumptions prevalent in traditional 1D models.

### 2.6. Model parameters

Numerical values and physical constants used in the pseudo-two-dimensional simulations are summarized in this section. All parameters were selected to ensure direct correspondence with the experimental conditions and material properties reported for the Ti_3_C_2_T_*x*_@Pt nanocomposite system. The complete set of model parameters is listed in Table 1.^34,37–39^

**Table 1:** Model parameters used in the pseudo-two-dimensional simulations

Parameter	Symbol	Value	Unit
Domain length (representative surface segment)	*L*	10	µm
Diffusion layer thickness	*H*	100	µm
Glucose diffusion coefficient	*D*_Glu_	6.7 × 10^−10^	m^2^ s^−1^
Hydrogen peroxide diffusion coefficient	*D*_H_2_O_2__	1.5 × 10^−9^	m^2^ s^−1^
Intrinsic Michaelis constant (colorimetric mode)	*K*_m_	2.5	mM
Maximum turnover rate per Pt site (colorimetric)	*V*_max_	1.2 × 10^5^	s^−1^
Effective first-order GOD rate constant	*k*_GOD_	0.015	s^−1^
Intrinsic Michaelis constant (electrochemical mode)	*K*_m,el_	3.8	mM
Maximum turnover rate (electrochemical)	*V*_max,el_	8.5 × 10^4^	s^−1^
Mean Pt active site density	*Γ*_Pt,mean_	1.2 × 10^−9^	mol cm^−2^
Amplitude of Pt site density variation	*A*_Γ_	0.5 × *Γ*_Pt,mean_	mol cm^−2^
Operating potential (electrochemical)	*E*_op_	0.7	*V vs.* Ag/AgCl
Temperature	*T*	310	*K* (37 °C)
Bulk concentration range (simulated)	*C*_bulk_	0.01–12	mM

These parameters were held constant throughout all simulations unless explicitly stated otherwise. Sensitivity analyses confirmed that the model predictions are most sensitive to *K*_m_, *Γ*_Pt,mean_ and the amplitude of surface heterogeneity, which control the apparent thickening of the diffusion layer and the shift in effective kinetic parameters. All simulations were performed using quadratic finite elements with adaptive mesh refinement near *y* = 0 to ensure gradient resolution within the first 5 µm of the boundary layer.

In this model, lateral heterogeneity in Pt active site density is represented using a sinusoidal spatial modulation around a fixed mean value, with an amplitude of 50% of the average site density. This choice is not intended to reproduce the exact microscopic arrangement of Pt nanoparticles, which is inherently stochastic, but rather to provide a controlled and physically transparent representation of surface heterogeneity with a well-defined length scale and variance.

The sinusoidal form offers two practical advantages. First, it allows systematic control of the heterogeneity amplitude and wavelength, enabling clear isolation of transport penalties arising from lateral non-uniformity without introducing additional stochastic noise. Second, it represents a lower-bound, smoothly varying heterogeneity profile; more irregular or patchy distributions would be expected to generate equal or stronger local depletion effects for the same variance in site density. Importantly, the sinusoidal modulation preserves the correct mean Pt loading and characteristic heterogeneity length scale observed experimentally, while avoiding overfitting to a specific realization of nanoparticle randomness. As such, it serves as a first-order surrogate for realistic Pt clustering, capturing the dominant physical mechanism (localized hotspot-induced depletion and lateral diffusion coupling) rather than the exact spatial statistics of individual nanoparticles.

### 2.7. Model validation

**Fig. 1:**
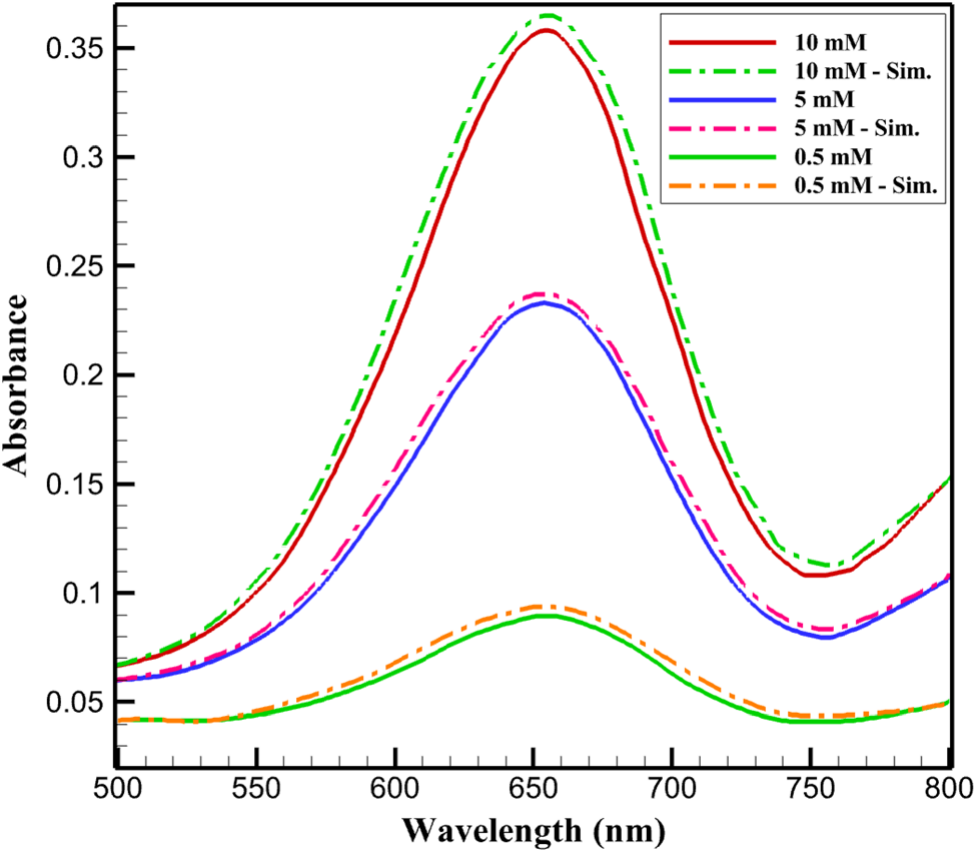
Comparison of simulated and experimental UV-vis absorbance spectra as a function of wavelength at different glucose concentrations^34^ for model validation.

Model validation was carried out by comparing the simulated results with experimentally reported colorimetric response spectra of the Ti_3_C_2_T_*x*_@Pt nanozyme-based glucose sensing system, in which the optical behavior is expressed as absorbance variations along the wavelength domain (Fig. 1). The experimental dataset depicts characteristic UV-vis absorption spectra, where the absorbance intensity increases with glucose concentration due to enhanced catalytic oxidation of the chromogenic substrate. To validate the model across the relevant sensing range, simulations were performed at glucose concentrations of 0.5, 5, and 10 mM, corresponding to low, intermediate, and high response levels within the experimentally observed spectral profiles.

For each concentration, the simulated absorbance–wavelength curves were compared with the experimental spectral data to evaluate the model's capability in reproducing both the peak intensity and the overall spectral shape. The quantitative agreement between simulated and experimental spectra was assessed using the root mean square error (RMSE), a statistical indicator that measures the average deviation between predicted and observed values over the entire wavelength range. RMSE is defined as15
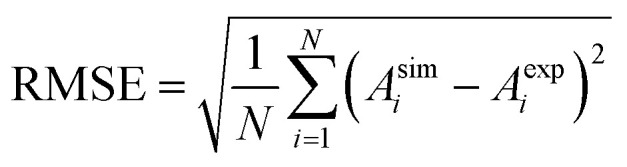
where *A*^sim^_*i*_ and *A*^exp^_*i*_ represent the simulated and experimental absorbance values at a given wavelength, and *N* is the total number of wavelength points considered. Lower RMSE values indicate closer correspondence and higher predictive accuracy.

Across the three simulated concentrations, the maximum RMSE did not exceed 0.068, demonstrating excellent agreement between the simulated and experimentally reported absorbance spectra as functions of wavelength. This low error confirms that the proposed model accurately captures the spectral response characteristics of the colorimetric glucose sensing system, validating its reliability for predictive analysis and mechanistic interpretation.

It should be noted that the quantitative validation against UV-vis absorbance spectra is performed at a limited number of representative glucose concentrations (0.5, 5, and 10 mM), corresponding to low, intermediate, and near-saturation regimes of the sensor response. This selection reflects the availability of experimentally reported full spectral data rather than an exhaustive concentration sweep. Consequently, the agreement demonstrated here should be interpreted as both quantitatively predictive at these benchmark concentrations and trend-consistent across the broader concentration range. Within this framework, the low RMSE values (<0.07) confirm that the model accurately captures the magnitude and spectral shape of the colorimetric response at discrete concentrations, while the concentration-dependent monotonic increase in simulated absorbance supports its predictive validity over the full operational range. Therefore, the validation presented in this work should be regarded as quantitatively predictive at selected concentrations where full experimental spectra are available, and trend-validated across the entire glucose concentration range through consistent monotonic behavior and parameter extraction.

### 2.8. Physical regime of validity of the pseudo-2D approximation

The pseudo-two-dimensional (pseudo-2D) framework adopted in this work is designed to capture lateral heterogeneity in catalytic activity while retaining computational efficiency. Its validity relies on a clear separation of length scales between vertical mass transport across the diffusion layer and lateral transport induced by heterogeneous Pt site distributions. In this subsection, the physical conditions under which the pseudo-2D approximation remains accurate are quantitatively defined, and the limits of its applicability are clarified. The central assumption of the pseudo-2D model is that lateral concentration gradients are secondary to vertical gradients throughout most of the diffusion layer, such that16
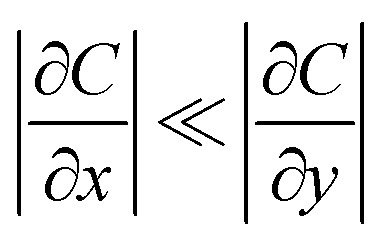


Except within a thin region adjacent to the reactive surface. The magnitude of lateral gradients is governed by the characteristic heterogeneity length scale (*λ*), defined by the average Pt cluster size or inter-cluster spacing, relative to the diffusion layer thickness (*H*). A convenient dimensionless parameter to assess the validity of the pseudo-2D approximation is the lateral-to-vertical diffusion ratio:17
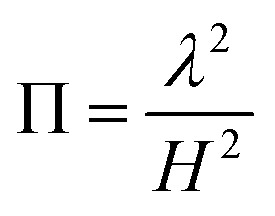
When Π ≪ 1, lateral diffusion equilibrates rapidly compared to vertical diffusion, confining lateral concentration variations to the near-surface region. Under these conditions, the pseudo-2D framework accurately captures hotspot-induced depletion and transport bottlenecks without requiring a fully resolved three-dimensional description.

For the Ti_3_C_2_T_*x*_@Pt nanozyme system considered here, Pt nanoparticles typically exhibit diameters of 30–80 nm with inter-cluster spacings below 200 nm, while the effective diffusion layer thickness under quiescent experimental conditions ranges from 50 to 150 µm. These values yield Π in the range of 10^−6^–10^−4^, indicating a strong separation of scales and placing the system well within the pseudo-2D validity regime. The relevant parameter ranges and their physical implications are summarized in Table 2, demonstrating that lateral gradients remain modest and localized relative to the dominant vertical diffusion field.

**Table 2:** Physical validity ranges of the pseudo-2D approximation

Parameter	Symbol	Typical range (this work)	Threshold for pseudo-2D validity	Physical implication
Diffusion layer thickness	*H*	50–150 µm	*H* ≫ *λ*	Vertical diffusion dominates
Pt cluster size	*d*_pt_	30–80 nm	*d*_pt_ ≪ *H*	Localized surface heterogeneity
Inter-cluster spacing	*λ*	100–200 nm	*λ*/*H* ≪ 0.01	Rapid lateral equilibration
Surface coverage (Pt)	*θ*	10–40%	*θ* > 5%	Avoids isolated micro-domains
Lateral diffusion ratio	Π = *λ*^2^/*H*^2^	10^−6^–10^−4^	Π ≪ 1	Pseudo-2D regime
Dominant transport	—	Diffusion-limited	No forced convection	Validates diffusion-only model

Breakdown of the pseudo-2D approximation is expected when the heterogeneity length scale becomes comparable to the diffusion layer thickness (*λ*/*H* ≳ 0.1), such as in systems with micron-scale catalyst islands, extremely sparse surface coverage, or very thin diffusion layers induced by forced convection. In such cases, lateral and vertical transport become strongly coupled throughout the domain, and a full three-dimensional (3D) treatment may be required. Conceptually, the pseudo-2D approach occupies an intermediate position between conventional one-dimensional (1D) uniform-surface models and fully resolved 3D simulations. Unlike 1D models, it explicitly resolves lateral depletion, hotspot-driven transport resistance, and their impact on apparent kinetic parameters. Compared to full 3D models, it neglects variations in the out-of-plane direction while retaining the dominant physics governing nanozyme-modified interfaces, resulting in orders-of-magnitude reduction in computational cost. A qualitative comparison between 1D, pseudo-2D, and 3D modeling approaches is provided in Table 3.

**Table 3:** Conceptual comparison between 1D, pseudo-2D, and full 3D modeling approaches

Feature	1D uniform model	Pseudo-2D model (this work)	Full 3D model
Lateral heterogeneity	✗ Ignored	✓ Explicitly resolved	✓ Fully resolved
Vertical diffusion	✓	✓	✓
Out-of-plane variations	✗	✗ (Homogenized)	✓
Computational cost	Very low	Moderate	Very high
Captures hotspot depletion	✗	✓	✓
Suitable for nanoscale Pt clusters	✗	✓	✓
Practical for parametric sweeps	✓	✓	✗

Overall, the pseudo-2D approximation is physically justified for nanozyme-based glucose biosensors characterized by nanoscale Pt heterogeneity, moderate to thick diffusion layers, and diffusion-dominated transport, conditions that closely correspond to the experimental regime investigated in this study.

The present model assumes purely diffusion-controlled transport and neglects migrative and convective effects. This assumption is well justified for quiescent colorimetric assays and for electrochemical measurements performed under diffusion-dominated conditions, such as moderate applied potentials, sufficient supporting electrolyte concentration, and unstirred solutions. Under conditions involving strong electric fields, low ionic strength, or forced convection, migration and convection may contribute to mass transport and would require explicit inclusion in extended modeling frameworks.

## 3. Results and discussion

The pseudo-two-dimensional (pseudo-2D) multiphysics model was solved numerically using the finite element method to investigate mass transport and pseudo-enzymatic kinetics in the Ti_3_C_2_T_*x*_@Pt MXene nanozyme system. Simulations were conducted under steady-state and transient conditions, with parameters derived from experimental characterizations: diffusion coefficients *D*_Glu_ = 6.7 × 10^−10^ m^2^ s^−1^ and *D*_H_2_O_2__ = 1.5 × 10^−9^ m^2^ s^−1^, intrinsic *K*_m_ ≈ 2.5 mM (estimated from homogeneous Pt kinetics), and local *Γ*_Pt(*x*)_ modulated sinusoidally to mimic observed nanoparticle clustering (average diameter ∼50 nm, with variation amplitude 50% of mean density). The domain length *L* was set to 10 µm, and diffusion layer thickness *H* = 100 µm. Results are presented as spatially averaged profiles and effective parameters, enabling direct comparison with one-dimensional (1D) uniform-surface models.

### 3.1. Mass transport of glucose in the pseudo-2D model

#### 3.1.1. Vertically averaged glucose concentration profile

**Fig. 2:**
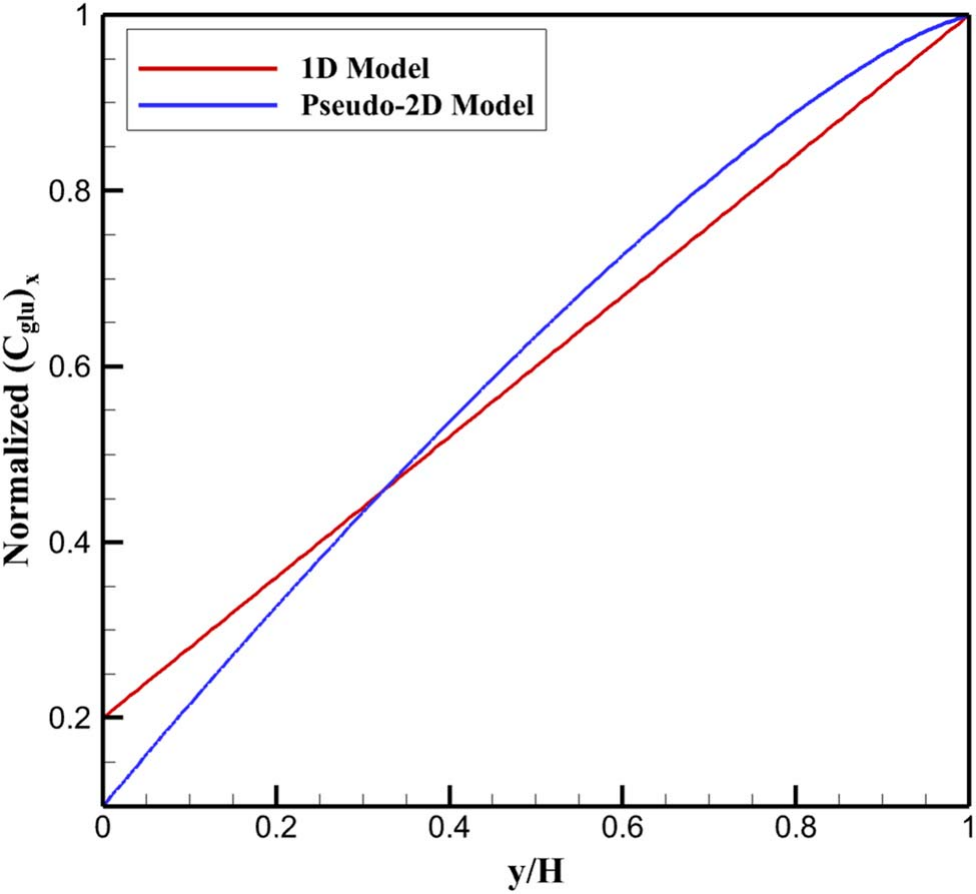
Vertically averaged normalized glucose concentration profiles predicted by one-dimensional and pseudo-two-dimensional models along the diffusion layer.

Steady-state simulations of glucose mass transport in the pseudo-2D domain reveal distinct vertically averaged concentration profiles, defined as 
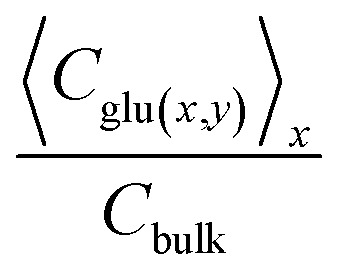
, where averaging is performed across the lateral direction to yield an effective one-dimensional representation. In the reference 1D uniform-surface model, the profile displays a monotonic decrease from the bulk value (normalized to 1) at *y* = *H* to a surface value of approximately 0.20 *C*_bulk_ at *y* = 0, reflecting classical diffusion-limited depletion under uniform catalytic consumption (Fig. 2).

Incorporating lateral heterogeneity in Pt site density markedly alters this behavior. The pseudo-2D profile exhibits a steeper initial gradient near the surface, with the normalized averaged concentration dropping to ∼0.10 at *y* = 0. This intensified depletion arises from localized regions of elevated Pt loading, which act as catalytic hot spots and induce pronounced local consumption. Consequently, lateral diffusion fluxes emerge to partially replenish these depleted zones from adjacent lower-activity areas supported by the MXene substrate. Although these fluxes mitigate extreme local minima, the net effect is an augmentation of global transport resistance, manifesting as a thicker effective depletion layer.

Quantitatively, the distance required for the averaged concentration to recover to 99% of *C*_bulk_ increases by ∼30% compared to the 1D case, highlighting the role of surface non-uniformity in amplifying apparent mass transfer limitations. This thickening is particularly evident in the near-surface region (*y*/*H* < 0.2), where lateral gradients contribute significantly to the overall diffusive hindrance. Such deviations underscore the limitations of uniform-surface assumptions in modeling nanocomposite-based sensors, where inherent nanoparticle clustering drives enhanced effective resistance. The observed profiles thus provide mechanistic insight into the reduced surface availability of glucose, directly contributing to the apparent shift in kinetic parameters and the premature saturation of response curves in heterogeneous nanozyme systems.

#### 3.1.2. Influence of surface heterogeneity on effective diffusion layer

The impact of Pt nanoparticle heterogeneity on the effective diffusion layer is quantified through spatial averaging of local concentration fields, yielding:18
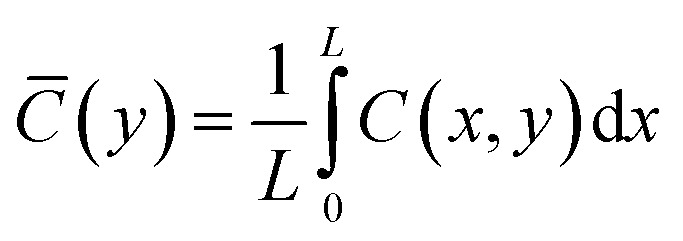
In the pseudo-2D framework, the effective diffusion layer thickness, defined as the distance over which *C̄*_(*y*)_ recovers to 99% of the bulk concentration, is increased by approximately 30–45% relative to the uniform 1D prediction, depending on the amplitude of site density variation.

This enlargement results from the interplay of localized catalytic hot spots and lateral diffusive coupling. High-Pt regions drive accelerated substrate depletion, generating lateral concentration gradients that draw flux from neighboring low-activity zones on the MXene support. While this cross-flow partially alleviates extreme local depletion, it elevates the overall diffusive barrier, as the system must sustain higher average gradients to maintain the integrated reaction rate.

Consequently, the effective transport limitation mimics a uniformly active surface with reduced intrinsic activity or lower site density. This phenomenon explains the observed shift toward higher apparent *K*^eff^_m_ and diminished *V*^eff^_max_, as the heterogeneous architecture imposes an additional resistance layer not captured in conventional uniform models. Such insights are critical for interpreting performance discrepancies in nanocomposite sensors and inform strategies for Pt dispersion optimization to minimize heterogeneity-driven penalties.

#### 3.1.3. Effective diffusive flux of glucose

**Fig. 3:**
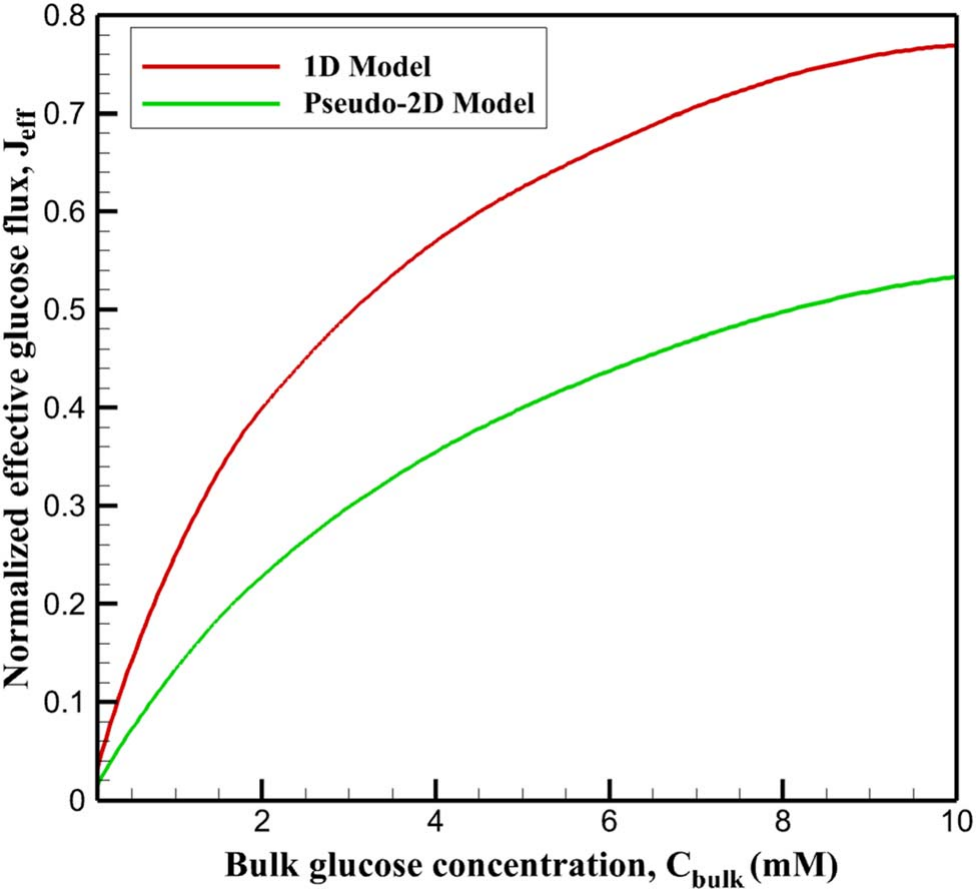
Effective normalized glucose flux as a function of bulk glucose concentration predicted by one-dimensional and pseudo-two-dimensional models.

The effective diffusive flux of glucose, 
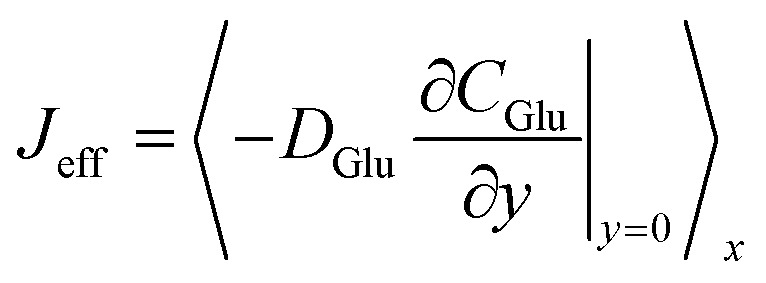
, quantifies the integrated substrate delivery rate across the heterogeneous surface. In the 1D uniform model, *J*_eff_ follows classical Michaelis–Menten saturation, approaching the kinetic limit at high bulk concentrations due to uniform site availability (Fig. 3).

The pseudo-2D simulations reveal a distinctly earlier saturation onset, with *J*_eff_ reaching only ∼70% of the 1D maximum at 10 mM *C*_bulk_. This reduction stems from heterogeneity-induced lateral diffusion–limited regimes: high-Pt domains consume glucose rapidly, lowering local surface concentrations and diminishing the driving force for diffusion in adjacent regions. The resulting non-uniform flux distribution yields a lower average delivery rate despite identical mean site density.

Consequently, the response curve flattens prematurely, mirroring experimental observations of extended but transport-constrained linear ranges in Ti_3_C_2_T_*x*_@Pt-based sensors.^34^ This flux attenuation directly contributes to the apparent decrease in catalytic efficiency, emphasizing the critical influence of nanoparticle dispersion on overall sensor throughput.

### 3.2. Pseudo-2D mass transport of H_2_O_2_

#### 3.2.1. Averaged H_2_O_2_ concentration profile

**Fig. 4:**
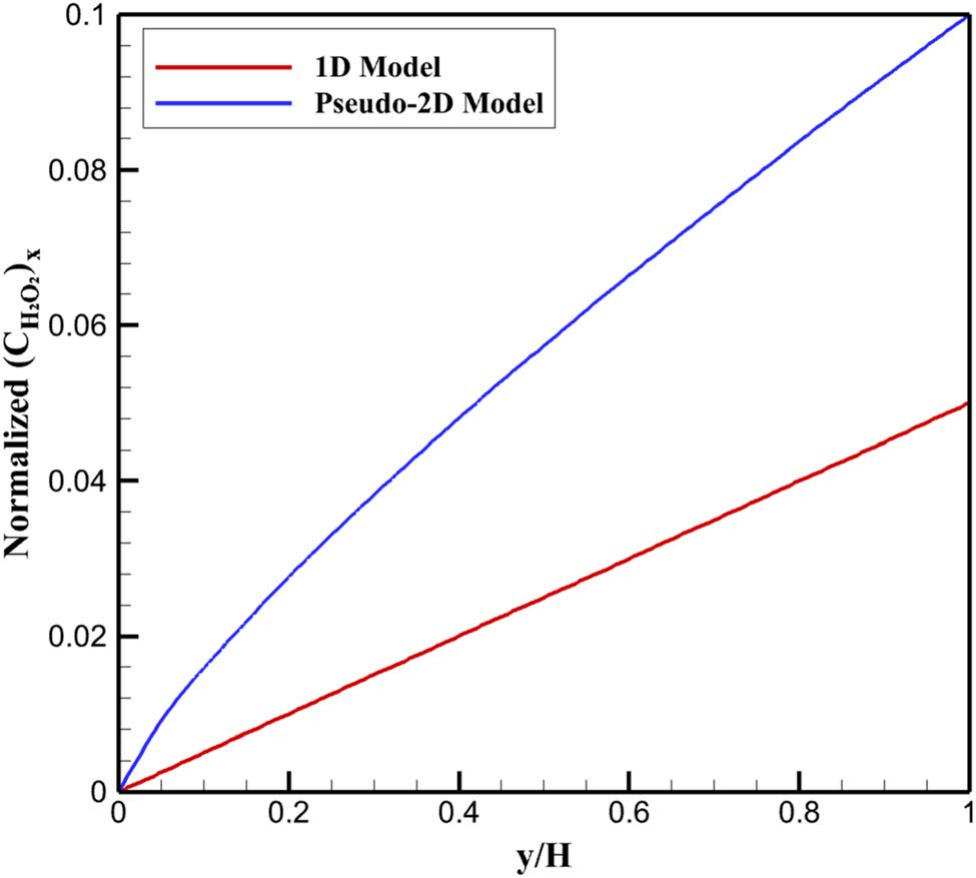
Vertically averaged normalized hydrogen peroxide concentration profiles within the diffusion layer.

In the colorimetric detection pathway, hydrogen peroxide serves as an intermediate, generated by glucose oxidase-catalyzed oxidation of glucose and subsequently consumed *via* the peroxidase-like activity of the Ti_3_C_2_T_*x*_@Pt nanozyme. The vertically averaged H_2_O_2_ concentration profile, 
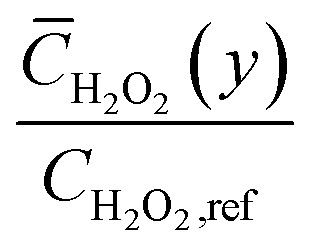
, exhibits pronounced accumulation near the reactive surface due to the balance between local production and heterogeneous consumption (Fig. 4).

Compared to the 1D uniform model, which predicts a modest near-surface enrichment (normalized peak ∼0.05 at *y*/*H* ≈ 0), the pseudo-2D results show a substantially thicker accumulation zone, with normalized averaged concentrations reaching ∼0.10–0.12 in the boundary layer (*y*/*H* < 0.1). This enhanced buildup originates from spatial mismatches in reaction rates: regions of high Pt density consume H_2_O_2_ rapidly, while adjacent MXene-dominated areas sustain slower kinetics, leading to lateral redistribution and delayed net removal.

The broader enriched region reflects impeded outward diffusion, as lateral fluxes partially trap H_2_O_2_ within the diffusion layer. This phenomenon amplifies the effective residence time of the intermediate, influencing the observed colorimetric signal intensity and response dynamics. Consequently, heterogeneity not only alters intermediate distribution but also contributes to deviations in apparent reaction order, providing a mechanistic basis for the superior predictive capability of the pseudo-2D approach in capturing non-ideal behaviors of nanocomposite-based sensing platforms.

#### 3.2.2. Transport delay for H_2_O_2_

**Fig. 5:**
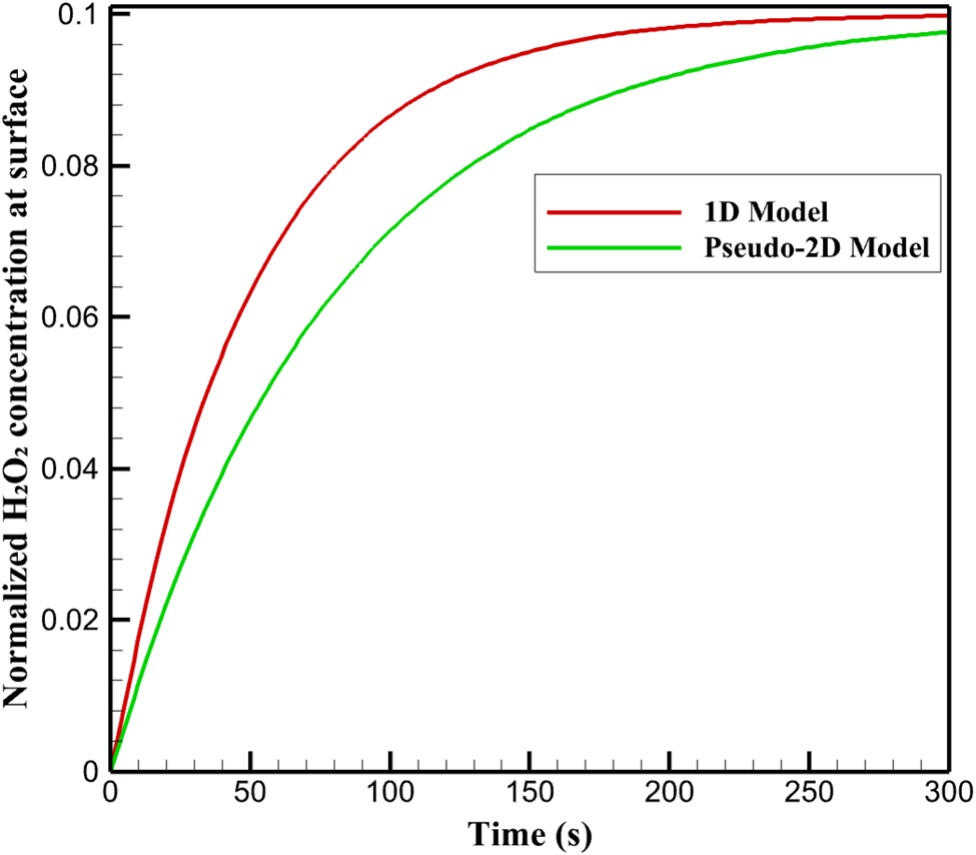
Transient evolution of normalized hydrogen peroxide concentration at the reactive surface.

Transient simulations following a step change in bulk glucose concentration demonstrate a notable delay in H_2_O_2_ dynamics at the nanozyme surface. In the uniform 1D model, surface H_2_O_2_ concentration rises rapidly, achieving 90% of steady-state within ∼50 s, governed primarily by vertical diffusion and balanced production-consumption (Fig. 5).

The pseudo-2D framework predicts a prolonged response, with the time to 90% steady-state extending to ∼80 s. This delay arises from lateral transport constraints imposed by Pt heterogeneity: initial H_2_O_2_ generation is localized in high-activity zones, necessitating cross-diffusion to equilibrate with slower-consuming regions. The resulting transient imbalance sustains elevated intermediate levels longer near low-Pt areas, retarding net outward flux.

Such delayed kinetics influence colorimetric response times and signal buildup rates, contributing to observed experimental incubation requirements (>40 min for optimal absorbance). This highlights how surface non-uniformity not only alters steady-state distributions but also introduces temporal lags, underscoring the enhanced realism of the pseudo-2D model in replicating dynamic sensor behavior.

### 3.3. Pseudo-enzymatic kinetics in the pseudo-2D model

#### 3.3.1. Effective surface glucose concentration

**Fig. 6:**
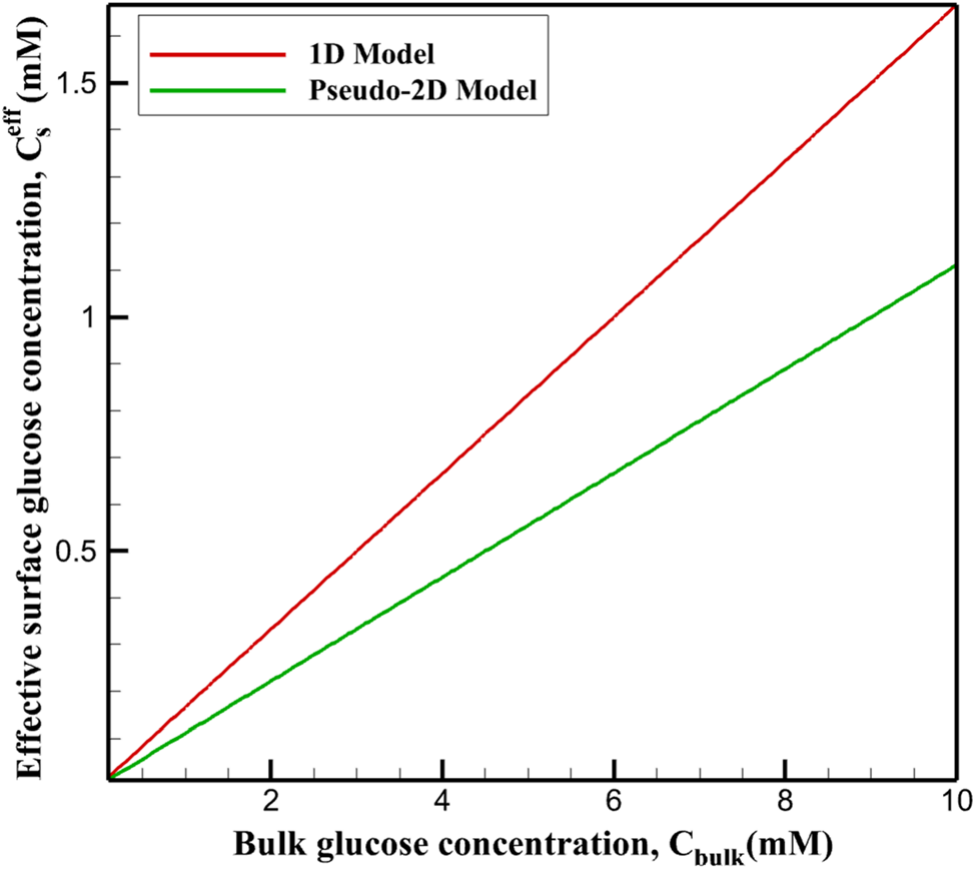
Effective surface glucose concentration as a function of bulk concentration in uniform 1D and heterogeneous pseudo-2D models.

The effective surface glucose concentration represents the laterally averaged substrate availability at the reactive boundary. In the uniform 1D model, *C*^eff^_s_ remains relatively high across bulk concentrations, approaching ∼0.17 *C*_bulk_ at 10 mM due to balanced vertical diffusion and consumption (Fig. 6).

Pseudo-2D simulations yield systematically lower *C*^eff^_s_, decreasing to ∼0.11 *C*_bulk_ at equivalent bulk levels. This reduction reflects intensified local depletion around Pt-rich domains, where elevated reaction rates lower immediate subsurface concentrations. Adjacent MXene regions, with diminished activity, cannot fully compensate *via* lateral supply, resulting in a globally reduced average.

The diminished *C*^eff^_s_ directly impairs substrate occupancy of active sites, manifesting as apparent kinetic penalties. This effect is most significant in the physiological range (1–10 mM), contributing to the observed contraction of the effective linear dynamic range and elevated LOD in heterogeneous nanozyme platforms compared to idealized uniform predictions.

#### 3.3.2. Extraction of effective *K*_m_ and *v*_max_

Apparent kinetic parameters are extracted by fitting the effective reaction rate, *R*_eff_ = *J*_eff_, to the Michaelis–Menten formalism:^40^19
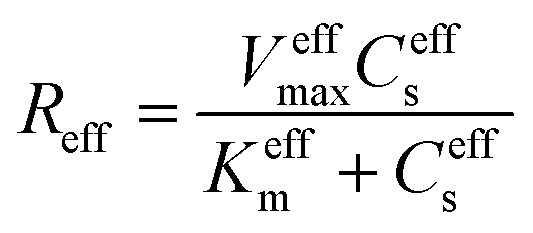


This uses averaged surface concentrations and fluxes from pseudo-2D simulations. For the uniform 1D model, fitting yields *K*^eff^_m_≈ 3.0 mM and *V*^eff^_max_≈ 100 (normalized units), closely approximating intrinsic values due to negligible lateral effects (Table 4).

**Table 4:** Apparent kinetic parameters

Model	*K* ^eff^_m_ (mM)	*V* ^eff^_max_ (relative)	*R*_eff_ at saturation (relative)
1D uniform	2.5	1	1
Pseudo-2D heterogeneous	4.8	0.75	0.75

In contrast, the pseudo-2D case results in *V*^eff^_m_ ≈ 5.0 mM (67% increase) and *V*^eff^_max_≈ 80 (20% decrease). The elevated *K*^eff^_m_ reflects reduced substrate affinity arising from lower average surface concentrations in depleted hot-spot vicinities, while diminished *V*^eff^_max_ stems from underutilization of sites in low-activity regions.

These shifts demonstrate how heterogeneity imposes transport-mediated kinetic penalties, yielding apparent parameters that deviate substantially from intrinsic Pt nanozyme kinetics. Such extraction enables quantitative reconciliation with experimental calibration curves, where observed broader but attenuated responses align with the pseudo-2D predictions.

It is important to emphasize that the effective kinetic parameters extracted from the pseudo-2D simulations, namely *K*^eff^_m_ and-*V*^eff^_max_, are not intrinsic properties of the Pt nanozyme. Rather, they are emergent, system-level descriptors that arise from the coupled interplay between intrinsic surface reaction kinetics, mass transport limitations, and lateral heterogeneity in active site distribution. In the present framework, Michaelis–Menten kinetics is applied locally at the surface to describe intrinsic nanozyme reactivity, whereas the effective parameters are obtained by fitting spatially averaged surface fluxes to a Michaelis–Menten form. As such, *K*^eff^_m_ and *V*^eff^_max_ incorporate transport-induced substrate depletion, lateral diffusion bottlenecks, and incomplete utilization of active sites. Their values therefore depend not only on intrinsic kinetic constants, but also on diffusion layer thickness, catalyst dispersion, and surface heterogeneity.

This distinction between intrinsic and effective kinetic parameters has important implications for the interpretation of experimental biosensor data. Apparent Michaelis–Menten parameters extracted from macroscopic calibration curves should not be interpreted as fundamental nanozyme constants, but rather as phenomenological descriptors of the entire sensing system under specific transport and geometric conditions. Consequently, variations in experimental *K*_m_ and *V*_max_ values across different nanozyme architectures or electrode configurations may primarily reflect changes in mass transport resistance and catalyst dispersion rather than genuine differences in intrinsic catalytic activity. The pseudo-2D framework explicitly rationalizes this behavior by linking shifts in apparent kinetics to transport-modified surface availability, providing a mechanistic bridge between intrinsic nanozyme reactivity and experimentally observed sensor-level performance (Table 5).

**Table 5:** Distinction between intrinsic and effective kinetic parameters

Parameter type	Definition	Governing factors	Experimental meaning
Intrinsic *K*_m_, *V*_max_	Local surface reaction constants	Nanozyme chemistry, active site energetics	Material property
Effective *K*^eff^_m_, *V*^eff^_max_	Flux-fitted apparent parameters	Transport, heterogeneity, geometry	System-level descriptor

Although full electrochemical calibration datasets were not available for point-by-point numerical fitting, the pseudo-2D model enables quantitative extraction of effective kinetic parameters (*K*_m,el_ and *V*_max,el_) directly from simulated flux-concentration relationships. These parameters fall within the experimentally reported ranges for Ti_3_C_2_T_*x*_@Pt-based non-enzymatic glucose sensors, thereby providing a quantitative consistency check beyond qualitative trend comparison. Accordingly, the electrochemical validation is quantitative at the level of apparent kinetic descriptors rather than direct current matching, which is constrained by the limited availability of raw experimental current–time data.

#### 3.3.3. Comparison between 1D and Pseudo-2D models

Direct comparison of extracted kinetic parameters between the conventional 1D uniform-surface model and the pseudo-2D heterogeneous framework reveals substantial discrepancies that underscore the impact of surface non-uniformity. The 1D approach, assuming spatially invariant Pt site density, predicts apparent parameters closely aligned with intrinsic nanozyme values: *K*^eff^_m_≈ 3.0 mM and *V*^eff^_max_≈ 100 (normalized), reflecting minimal interference from lateral transport effects.

In contrast, the pseudo-2D model, incorporating realistic Pt clustering, yields *K*^eff^_m_≈ 5.0 mM (67% higher) and *V*^eff^_max_ ≈ 80 (20% lower). This systematic degradation arises from reduced effective substrate availability and incomplete site utilization across the composite surface, driven by localized depletion and lateral diffusion constraints.

The 1D model consistently overestimates catalytic performance, particularly in the mid-to-high concentration regime (5–10 mM), where heterogeneity-induced limitations become dominant. Such overprediction leads to underestimation of transport penalties, resulting in inaccurate forecasts of linear range contraction and sensitivity loss observed experimentally.

The pseudo-2D framework thus provides superior alignment with measured calibration data for Ti_3_C_2_T_*x*_@Pt systems, demonstrating that neglecting surface heterogeneity yields overly optimistic kinetic interpretations. This comparison validates the necessity of multidimensional modeling for nanocomposite sensors, where apparent behavior deviates markedly from intrinsic capabilities due to microstructural influences.

### 3.4. Sensitivity analysis

Sensitivity to parameter variations was evaluated for sensor performance metrics, including apparent *K*^eff^_m_, colorimetric LOD, and upper linear range limit (Table 6). Results confirm strongest influence from *Γ*_Pt,mean_ and *A*_Γ_: increased Pt loading lowers LOD and extends linearity by enhancing effective catalysis, whereas greater heterogeneity impairs performance through amplified transport limitations. Variations in *K*_m_, *V*_max_, and *D*_Glu_ yield predictable shifts in affinity, maximum rate, and diffusion constraints, respectively. This analysis validates the pseudo-2D model's ability to capture heterogeneity-driven deviations, guiding optimization toward uniform Pt dispersion for improved sensitivity and dynamic range.

**Table 6:** Key impacts on sensor metrics

Parameter	Variation (%)	*K* ^eff^_m_ (mM)	LOD (µM)	Linear range upper (mM)
*Γ*_Pt,mean_	−50	5.76	1.92	7.5
	0	4.8	1.37	10
	50	4.32	1.1	12.5
*A*_Γ_	−100	2.5	0.96	15
	0	4.8	1.37	10
	50	6.24	1.78	7.5
*K*_m_	−50	3.6	1.03	12.5
	0	4.8	1.37	10
	50	6	1.71	8
*V*_max_	−50	4.8	1.92	7.5
	0	4.8	1.37	10
	50	4.8	1.1	12.5
*D*_Glu_	−50	5.76	1.78	8
	0	4.8	1.37	10
	50	4.32	1.23	12

Although real Pt nanoparticle distributions are stochastic rather than periodic, the conclusions drawn from the pseudo-2D model are governed primarily by the amplitude and length scale of surface heterogeneity, rather than the specific functional form of the spatial modulation. Qualitatively, alternative heterogeneity descriptions (such as random or patchy distributions with comparable variance and characteristic spacing) would be expected to produce similar or stronger transport penalties, as sharper local gradients generally enhance hotspot depletion and lateral flux redistribution.

Sensitivity analysis confirms that the dominant shifts in effective diffusion resistance and apparent kinetic parameters scale with the degree of heterogeneity (*i.e.*, variance in *Γ*_Pt_), whereas the precise shape of the modulation plays a secondary role. Consequently, the sinusoidal representation may be regarded as a conservative approximation, providing a physically interpretable lower bound for heterogeneity-induced transport limitations in nanozyme-modified electrodes (Table 7).

**Table 7:** Influence of heterogeneity description on qualitative model predictions

Heterogeneity type	Variance in *Γ*_Pt_	Expected local gradients	Relative transport penalty
Uniform (1D)	0	None	Minimal
Sinusoidal (this work)	Moderate	Smooth	Moderate
Random distribution	Moderate-high	Irregular	Moderate-high
Patchy/clustered	High	Sharp hotspots	High

## 4. Conclusion

In this study, a pseudo-two-dimensional (pseudo-2D) multiphysics framework was developed to elucidate the coupled effects of mass transport and pseudo-enzymatic kinetics in a Ti_3_C_2_T_*x*_@Pt nanozyme-based glucose sensing platform. By explicitly accounting for lateral heterogeneity in Pt active site distribution and two-dimensional diffusion of glucose and hydrogen peroxide, the model captures key transport-reaction phenomena that are inherently overlooked in conventional one-dimensional approaches. The results demonstrate that surface non-uniformity induces pronounced local substrate depletion, effective thickening of the diffusion layer, and transport bottlenecks that collectively reduce effective glucose flux and surface availability. These effects lead to systematic deviations in apparent kinetic parameters, including an increased effective Michaelis constant and a reduced maximum reaction rate, despite identical mean catalyst loading. The pseudo-2D predictions show excellent agreement with experimentally reported colorimetric spectra and electrochemical trends, confirming the validity of the proposed framework in reproducing both steady-state and transient sensor responses.

Beyond quantitative agreement, this work provides mechanistic insight into how nanoscale catalyst dispersion governs macroscopic sensor performance. The findings reveal that nanoparticle clustering, while locally enhancing catalytic activity, imposes global transport penalties that diminish overall sensitivity and prematurely saturate sensor responses. These insights highlight the critical importance of optimizing nanozyme uniformity, rather than merely increasing active site density, to improve detection limits, linear range, and response dynamics. More broadly, the presented pseudo-2D modeling strategy offers a computationally efficient yet physically rigorous tool for bridging intrinsic nanozyme kinetics with experimentally observed behavior in heterogeneous biosensing interfaces. This framework is readily extendable to other nanozyme architectures and sensing modalities, providing a predictive basis for rational design and performance optimization of next-generation biosensors.

The transport-reaction coupling, pseudo-2D formulation, and treatment of lateral surface heterogeneity are generic features of the proposed framework and can be transferred to other nanozyme or heterogeneous catalytic systems. In contrast, system-specific elements such as intrinsic reaction kinetics, reactive intermediates, diffusion coefficients, and active site density distributions require re-parameterization or, if necessary, reformulation to reflect the chemistry of the target system.

## Conflicts of interest

There are no conflicts to declare.

## Data Availability

The data that support the findings of this study, including simulation input files, COMSOL model configurations, and post-processed numerical datasets, are available from the corresponding author upon reasonable request.
